# Standard reference values of the upper body posture in healthy male adults aged between 31 and 40 years in Germany-an observational study

**DOI:** 10.1186/s40101-021-00266-w

**Published:** 2021-10-29

**Authors:** Daniela Ohlendorf, Ugur Kaya, Julian Goecke, Gerhard Oremek, Hanns Ackermann, David A. Groneberg

**Affiliations:** 1 Institute of Occupational Medicine, Social Medicine and Environmental Medicine, Goethe-University Frankfurt/Main, Theodor-Stern-Kai 7, Building 9A, 60590 Frankfurt/Main, Germany; 2 Institute of Biostatistics and Mathematical Modeling, Goethe-University, Frankfurt/Main, Theodor-Stern-Kai 7, Building 11A, 60596 Frankfurt/Main, Germany

**Keywords:** Upper body posture, Healthy men, Video rasterstereography, Standard value, BMI

## Abstract

**Background:**

In order to classify and analyze the parameters of upper body posture, a baseline in the form of standard values is demanded. To this date, standard values have only been published for healthy men aged 18–35 and 41–50 years. Data for male adults aged between 31 and 40 years are lacking.

**Methods:**

The postural parameters of 101 symptom-free male volunteers aged 31–40 (35.58 ± 2.88) years were studied. The mean height of the men was 179.89 ± 7.38 cm, with a mean body weight of 86.36 ± 11.58 kg and an average BMI of 26.70 ± 3.35 kg/m^2^. By means of video rasterstereography, a 3-dimensional scan of the upper back surface was measured in a habitual standing position. The means or medians, confidence interval, tolerance range, and group comparisons and correlations of BMI and physical activity were calculated for all parameters.

**Results:**

The habitual standing position was found to be almost symmetrical and the axis aligned in the spine, pelvis, and shoulder region, while the spine position was marginally inclined ventrally. The kyphosis angle of the thoracic spine was greater than the lordosis angle of the lumbar spine. All deviations fell under the measurement error margin of 1 mm/1°. The greater the BMI, the greater was the pelvic and scapular distance. The lower the BMI, the further caudally positioned was the right shoulder. The pelvic and scapular distances were also lower with the increasing athleticism of the participants.

**Conclusion:**

The upper body posture of men between the ages of 31 and 40 years was found to be almost symmetrical and axis-conforming, with the kyphosis angle, pelvic distance, and shoulder distance enlarging with increasing BMI. Consequently, postural parameters presented in this survey allow for comparisons with other studies, as well as the evaluation of clinical diagnostics and applications.

## Background

Most health diseases in modern industrialized nations include back pain [1]. ^(p10)^

This is also coded in the current version 10 of the International Statistical Classification of Diseases and Related Health Problems (ICD 10) under Chapter XIII “Diseases of the musculoskeletal system and connective tissue” (M00-M99) in section M.54 “Diseases of the spine and back (M40-M54)” [2].

Back pain summarizes various complaints of the entire back (cervical, thoracic and lumbar spine), which are, however, differentiated according to their cause or trigger, type, and severity. Back pain can result from, among other things, asymmetrical muscular strain (muscular imbalances) or tension that becomes visible through poor posture. It can also be caused by occupational activities, such as lifting and carrying heavy loads in unsuitable postures, forced postures, or monotonous postures to a much greater extent than is the case in the rest of the population [3].

In Germany, 22.5% of people between 20 and 75 years of age suffer from persistent back pain [4] and it is, thus, one of the main causes of incapacity to work [1]. In 2017, the Federal Ministry of Health registered 2025 cases of incapacity to work, with 196.711 working days lost due to incapacity and an average incapacity to work of 97.14 days per case due to back pain [5].

With regard to existing back pain, a prevalence of 29.1% [6] has been determined for men in Europe. Concerning men in the age group of 31–40 years, the following figures have been recorded for Germany: those aged 30–34 years were unable to work for an average of 13.07 days per case due to musculoskeletal disorders, while for men aged 35-39 years, this was 14.79 days per case [7]. Of these, 23.9 days for men aged 30–35 years and 23.8 days for men aged 35–40 years were spent in inpatient rehabilitation facilities due to musculoskeletal disorders [8]. Therefore, this results in a high loss of working days for men aged 30-40 years [1]. Considering these data, young employees between 31 and 40 years of age are relatively often affected by back pain. Therefore, there is a need for action to ensure that young, employed men remain employable for at least 35 years due to their young age and their low level of physical and mental decline.

In addition, these men represent a group of people whose physical integrity should be protected by their low level of mental and physical decline and, thus, should be able to remain in the labor market in the longer term.

In order to understand better the correlation between upper body posture and back pain, it is essential to be in the possession of standard values of symptom-free people, grouped by age and sex. With the help of these data, deviations from the physiological normal state can be easier to distinguish and recognize and can be used to help deciding on a therapy.

Studies that differentiate sex- and age-specific standard reference values of the upper body to realize uniform assessment standards have already been published for healthy male test persons between 18–35 years [9] and 41–50 years [10] of age, as well as for healthy female test participants between 20–30 years [11] and 51–60 years [12] of age. The aims of these individual publications are to measure the upper body posture in dependence of age and sex of the working population in Germany via video raster stereography which are part of a project and is described in the methodology paper by Ohlendorf et al. [13] In addition to X-rays, there are other methods for documenting the spine which, in contrast to X-rays, are radiation-free and, therefore, suitable for frequent documentation. These include the video raster stereography method, in which the back surface can be displayed three-dimensionally using light projections. This method [14] has high (intra- and inter-day) reliability and reproducibility while its accuracy is increased with the use of given anatomical landmarks [15].

Therefore, the aim of the present study is (i) to generate standard values of upper body posture for the male age group of 31–40 years and to classify them in previously published data and (ii) to analyze whether BMI or physical activity has an effect on upper body posture. The following hypotheses are to be tested:

1. Present standard values are in agreement with previous analyses (men aged 18–35 years and 41–50 years) regarding sagittal and frontal parameters.
2. The higher the BMI, the more pronounced sagittal parameters are.
3. Men who engage in sports have a more upright posture, in terms of frontal trunk inclination and axis deviation closer to the 0° axis.

## Methods

### Subjects

This study is part of the standard reference value project that focuses among others on the upper body posture of healthy adults and has already been published in parts earlier [9–13].

In the present study, 101 male subjects within the age decade of 31 to 40 years (35.58 ± 2.88 years) voluntarily participated. Further constitutional parameters are listed in Table 1.

**Table 1:** Presentation of medians/averages, tolerance ranges, and confidence intervals with associated lower and upper, left and right limits in relation to height, body weight, and BMI

Parameter	Mean value/median	Tolerance range		Confidence interval	
		Lower limit	Upper limit	Left limit	Right limit
Body height (cm)	179.89	165.17	194.61	178.43	181.35
Body weight (kg)	85.00	64.55	110.90	82.00	90.00
BMI (kg/m^2^)	26.20	21.00	34.78	25.42	27.00

Body mass index (BMI, kg/m^2^) was differentiated according to the WHO classification [8, 16, 17] as follows: 12 subjects (11.88%) were in the normal BMI range (18.5–24.9 kg/m^2^), 72 subjects (71.29%) were pre-adipose (25-29.9 kg/m^2^), and 17 subjects (16.83%) were obese (≥30 kg/m^2^).

All participants reported their health subjectively; those with musculoskeletal, spine, shoulder, and pelvic conditions, limitations, pain, and interventions were excluded from participation. “Healthy” means that the subjects have no acute symptoms and subjectively described themselves as healthy at the time of measurement. Furthermore, subjects who were undergoing physiotherapy or orthopedic treatment at the time of the study, or who were taking muscle relaxants, or had evidence of poor posture, as determined by a doctor, were prohibited from participating. Another reason for exclusion was surgery that had taken place within the last 2 years.

Before the start of the study, all subjects were fully informed about the modalities of the study and a written informed consent was obtained.

At the beginning, anamnestic parameters, such as age, height, and weight, as well as questions about general illnesses and general sporting activity were recorded [18].

The evaluation revealed that 30.70% of the participants were employed in an office, 51.48% of the participants had a non-office job. Among the participants without an office-job, there were cooks, dentists, hair stylists, firemen, bakers, and handymen for example. Moreover, 17.82% of the participants did not give any information about their occupation. While 46,53% of the test persons were physically inactive around 53.47% of the selected participants regularly engaged in sports in their leisure time.

An approved ethics application was submitted for the conduct of the study (ethics no.: 103/16). Its specifications refer to the ethical principles for medical research on humans. These are set out in the current version of the Declaration of Helsinki from 2013.

### Measurement system: back scan

**Fig. 1:**
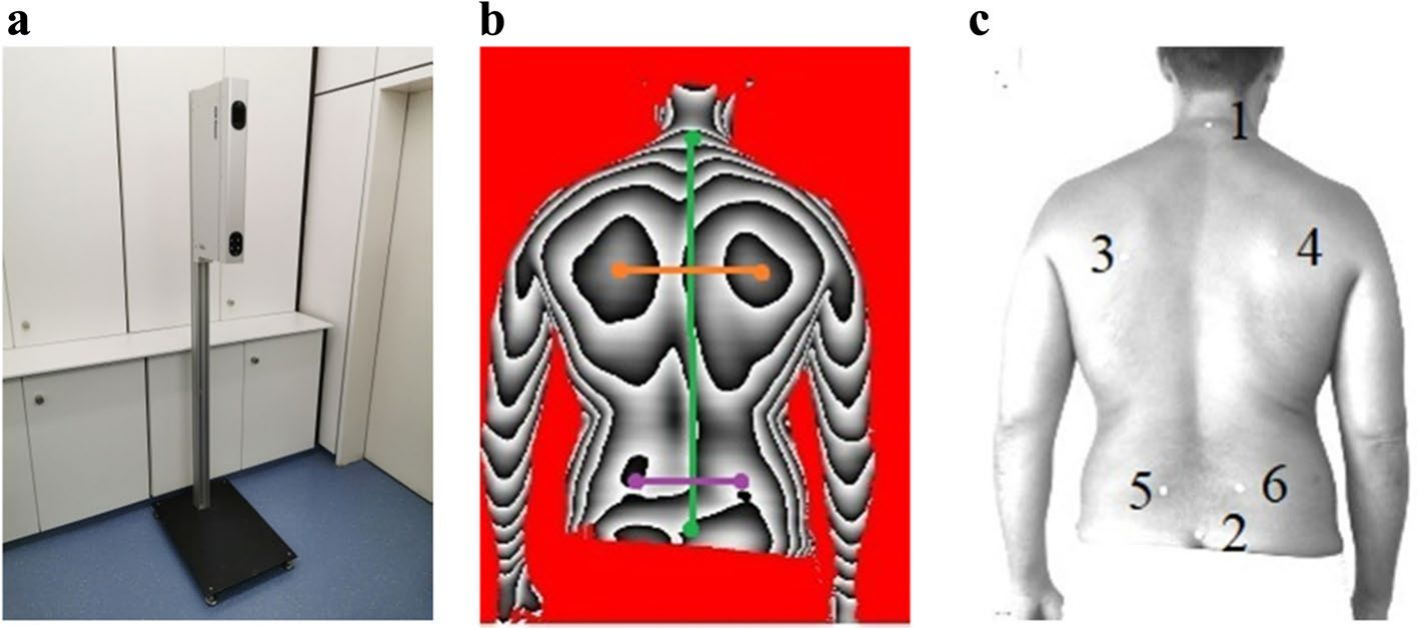
**a** Illustration of the 3D-back scan. **b** Representation of the gray-scale image; color coding of the lines: green = spine, orange = shoulder, purple = pelvis. **c** Display of the contour line image; (1) VP, (2) SP, (3) AISL, (4) AISR, (5) DL, (6) DR

The mobile three-dimensional back scanner “Bodymapper” (ABW GmbH, Frickenhausen, Germany; Gebiom, Münster, Germany) was used to illuminate the back (Fig. 1a). The three-dimensional back scanner projects a striped pattern onto the unclothed upper body of the test person via a light projection for light-optical measurement; this measurement was registered via a camera from a fixed angle (video raster stereography). The maximum frame rate was 50 frames/s with a depth resolution of 1/100 mm, with 15 video frames recorded per second. Using a dynamically moving strip light (triangulation technology), the light-irradiated fixed points were determined and graphically mapped in 3D coordinates [11, 19]. For this purpose, a height image (Fig. 1b) and a grayscale image (Fig. 1c) were generated.

According to the manufacturer, the measurement error is less than 1 mm. A reproducible measurement accuracy of 0.5 mm has been achieved with recurring measurements [20].

For the examination, the test persons were asked to undress their upper body up to and including the beginning of the buttock fold and to stand on the floor at a distance of 90 cm from the scanner with their backs facing the scanner on a defined line. In order to achieve the greatest possible accuracy and to minimize interfering factors, barefoot measurement was insisted on. Furthermore, the test persons were asked to fix their gaze at eye level and to adopt a habitual posture for the duration of the measurement.

Beforehand, six defined fixed points on the back were marked with adhesive markers [11]:

(VP) Vertebra prominens (7th cervical vertebra)
(SP) Point at Os sacrum (beginning of the gluteal fold "Rima ani")
(AISL) Shoulder blade angle left
(AISR) Shoulder blade angle right
(DL) Spina iliaca posterior superior left
(DR) Spina iliaca posterior superior right

Subsequently, 3 measurements with a recording duration of 0.6 s each were carried out, whereby each evaluation parameter was averaged for further statistical analysis as follows.

#### Evaluation parameter

Points 1–2 represent the spine region (green), points 3–4 the shoulder region (orange), and points 5–6 the pelvic zone (purple), as illustrated in Fig. 1b, c.

Thus, a total of 25 parameters of the upper body were determined in the spine, shoulder and pelvis areas and calculated using the manufacturer's software (BackMapper, ABW GmbH, Frickenhausen, Germany). A detailed explanation of the parameters can be found in the method paper [13] and in Table 2.

**Table 2:** Spine, shoulder, and pelvis parameters: mean values, medians, tolerance ranges (upper and lower limits), and confidence intervals (left and right limits). Lines with gray background are non-parametrical data

	Mean value/median	Tolerance range Lower limit	Tolerance range Upper limit	Confidence interval Left limit	Confidence interval Right limit
**Spine parameter**					
Trunk length D (mm) Spatial distance between the markers C7 and middle of the PSIS-marker	473.95	414.18	533.72	468.03	479.87
Trunk length S (mm) Spatial distance between the markers at C7 and Rima Ani	526.03	470.21	582	520.57	531.64
Sagittal trunk decline (°) Inclination of the trunk length D marked line from the perpendicular to the sagittal plane	− 2.63	− 8.46	3.19	− 3.21	− 2.06
Frontal trunk decline (°) Inclination of the trunk length D marked line from the perpendicular to the frontal plane	− 0.34	− 3.57	2.04	− 0.56	− 0.07
Axis decline (°) Deviation of the line of the area marked by the trunk length D line of the 90 ° rotated distance between PSIS left and PSIS right	− 0.98	− 5.16	3.19	− 1.40	− 0.57
Thoracic bending angle (°) Deviation of the distance C7 – Kyphosis Apex from the perpendicular	15.66	7.99	23.32	14.90	16.41
Lumbar bending angle (°) Deviation of the distance Kyphosis Apex – Lordosis Apex from the perpendicular	10.99	5.49	16.50	10.45	11.54
Standard deviation lateral deviation (°) Root mean squared deviation of the median line of the distance C7 – center of the PSIS marker	4.70	1.49	11.20	3.72	5.11
Standard deviation rotation (°) Root mean square deviation of surface rotation of the median line (torsion of the spinous processes of the spine)	3.41	1.20	11.65	2.95	4.16
Kyphosis angle (°) Angle between the upper turning point at C7 and the thoracolumbar inflection point	52.56	34.31	70.82	50.76	54.37
Lordosis angle (°) Angle between the lower inflection point at the center of the PSIS marker and the thoracolumbar turning point	32.16	17.25	47.08	30.69	33.64
**Shoulder parameter**					
Scapular distance (mm) Distance between the left (AISL) and the lower right scapular angle (AISR)	185.45	138.28	232.63	180.78	190.12
Scapular height (°) Height difference between the AISL and AISR points	− 4.35	− 17.76	10.25	− 5.14	− 2.37
scapular rotation (°) Rotation of the distance AISL - AISR in the transversal plane	0.77	− 5.42	6.96	0.16	1.38
Left scapular angle (°) Angle of the compensation line applied from the shoulders to the horizontal. The center of the compensation line is specified vertically above AISL	26.60	16.92	45.10	25.47	27.86
Right scapular angle (°) Angle of the compensation line applied from the shoulders to the horizontal. The center of the compensation line is specified vertically above AISR	27.93	15.37	45.12	26.60	29.43
**Pelvis parameter**					
Pelvic distance (mm) Spatial distance between the left (PSISL) and right (PSISR) of the pelvis	127.42	92.85	162.00	124.00	130.85
Pelvic height (°) Decline of the connecting line between PSIS left and PSIS right to the horizontal in the frontal plane in degrees	− 0.46	− 4.25	3.33	− 0.84	− 0.09
Pelvic height (mm) Decline of the connecting line between PSIS left and PSIS right to the horizontal in the frontal plane in millimeter	− − 1.25	− 8.67	7.76	− 2.34	− 0.56
Pelvic torsion (°) PSIS left – PSIS right, twist around the transverse axis calculated from the mutual twisting of the surface normal on the two PSIS	1.16	− 11.60	13.92	− 0.10	2.42
Pelvic rotation (°) Rotation of the distance PSIS left – PSIS right in the transversal plane	0.32	− 7.11	7.75	− 0.42	1.06

#### Statistical evaluation

All calculations were carried out using BIAS (Version 11.0) (Epsilon Verlag, Darmstadt, Germany). The parameter distribution was tested by the Kolmogorov-Smirnov test which indicated only partial normal distribution; parametrical or non-parametrical tolerance regions (TR) were calculated as defined by the upper and lower limits for 95% of all values (±2 SD values) being found in > 95% of the examined subjects. Values within this range were considered “normal”.

Furthermore, the two-sided 95% confidence interval (left limit/right limit CI) was calculated and indicated the range of the mean or median value (depending on the distribution quality) and showed the “accuracy” of these values. For group differences, a Kruskal-Wallis test was used followed by a pairwise comparison (Conover-Iman test). All *p* values were corrected by Bonferroni-Holm.

The calculation of the correlations was conducted by using Spearman-Kendall correlations. The effect strength served to evaluate the correlation coefficient rho according to Evans and was defined as follows: 1 = < 0.2, poor; 2 = 0.2–0.4, weak; 3 = 0.4–0.6, moderate; 4 = 0.6–0.8, strong; 5 = > 0.8, optimal.

## Results

Table 2 contains the mean and median values, the tolerance range (TR) with its upper and lower limits, and the confidence interval with its left and right limits of all measured parameters.

On average, the male subjects aged between 31 and 40 years stood slightly inclined in the anterior line by 2.63° (tolerance range 8.46° ventrally to 3.19° dorsally; confidence range 3.21° to 2.06° ventrally).

In the frontal plane, there was a minimal deviation of the trunk of 0.34° to the left, the CI the CI ranged from − 0.56° (left limit) to − 0.07° (right limit) and the TR ranged from − 3.57° to the left to 2.04° to the right. The axial deviation (inclination between the upper body and pelvis) was also slightly tilted to the left (0.98°), with a tolerance range of − 5.16° (lower limit) and 3.19° (upper limit) and a confidence interval of between − 1.40° and − 0.57° to the left.

The thoracic bending angle (calculated from the distance between VP and the kyphosis apex) also indicated this deviation from the perpendicular line and confirmed a thoracic kyphosis with an angle of 15.66°. Here, variations of the TR ranged from 7.99° (upper limit) to 23.32° (lower limit) and the CI varied between 14.90° (left limit) and 16.41° (right limit). Compared to the thoracic bending angle, similar variations of the TR and the CI were seen for the lumbar bending angle (deviation of the distance between the lordosis and kyphosis apex), having a bending angle of 10.99° (TR lower limit 5.49°, upper limit 16.50°; CI left limit 10.45°, right limit 11.54°). Same relationship (kyphosis angle > lordosis angle) count for the kyphosis and lordosis angles, having a mean or a median of 52.56° and 32.16°, with a substantial TR of approximately ± 21° and ± 17° and a CI of about ± 2°.

The median lateral deviation showed a slightly right-sided inclination by 4.70° when combining VP and the center of the pelvic markers. Both TR (1.49° and 11.20°, respectively) and CI (3.72°/5.11°) also indicated a right-sided deviation. The rotation of the spinal column indicates the spinal column torsion, measured from the spinal processes: here, a negative value indicates a rotation to the left and a positive value a rotation to the right. The median spinal rotation in the study was right-sided with a value of 3.41°, with a TR of between 1.20° and 11.65° and a CI of between 2.95° and 4.16°.

The lower scapular spinae were measured by two fixed markers. The distance between the two scapular markers indicated the variable shoulder width which was 185.45 mm (mean), with a TR of 138.28–232.63 mm and a CI limit of 180.78–190.12 mm. The scapular height indicates the left shoulder deviation from the horizontal line being more caudally by 4.35° (TR: lower limit − 17.76°, upper limit 10.25°; CI left limit − 5.14°, right limit − 2.37°). The same was true for the shoulder blade angle left and right, whereas the left shoulder was located more caudally than the right. The shoulder markers illustrated the right shoulder to be slightly further dorsal by 0.77°, with a tolerance range of − 5.42° to 6.96° and a CI of 0.16° to 1.38°.

In terms of the pelvic parameters, the pelvis width was referred to as the distance between the spina iliaca posterior superior markers, which, on average, was 127.42 mm (TR of 92.85 to 162.00 mm; CI of 124.00 and 130.85 mm). The pelvic height (horizontal plane) was almost balanced. Both differences in millimeters and degrees indicate a slightly higher position of the left pelvis by a median of 0.46° or 1.25 mm. In contrast, the pelvic torsion and rotation show that the right iliac marker was rotated posteriorly and simultaneously tilted further ventrally (mean pelvis torsion: 1.16°; mean pelvic rotation: 0.32°).

### Body mass index

**Fig. 2:**
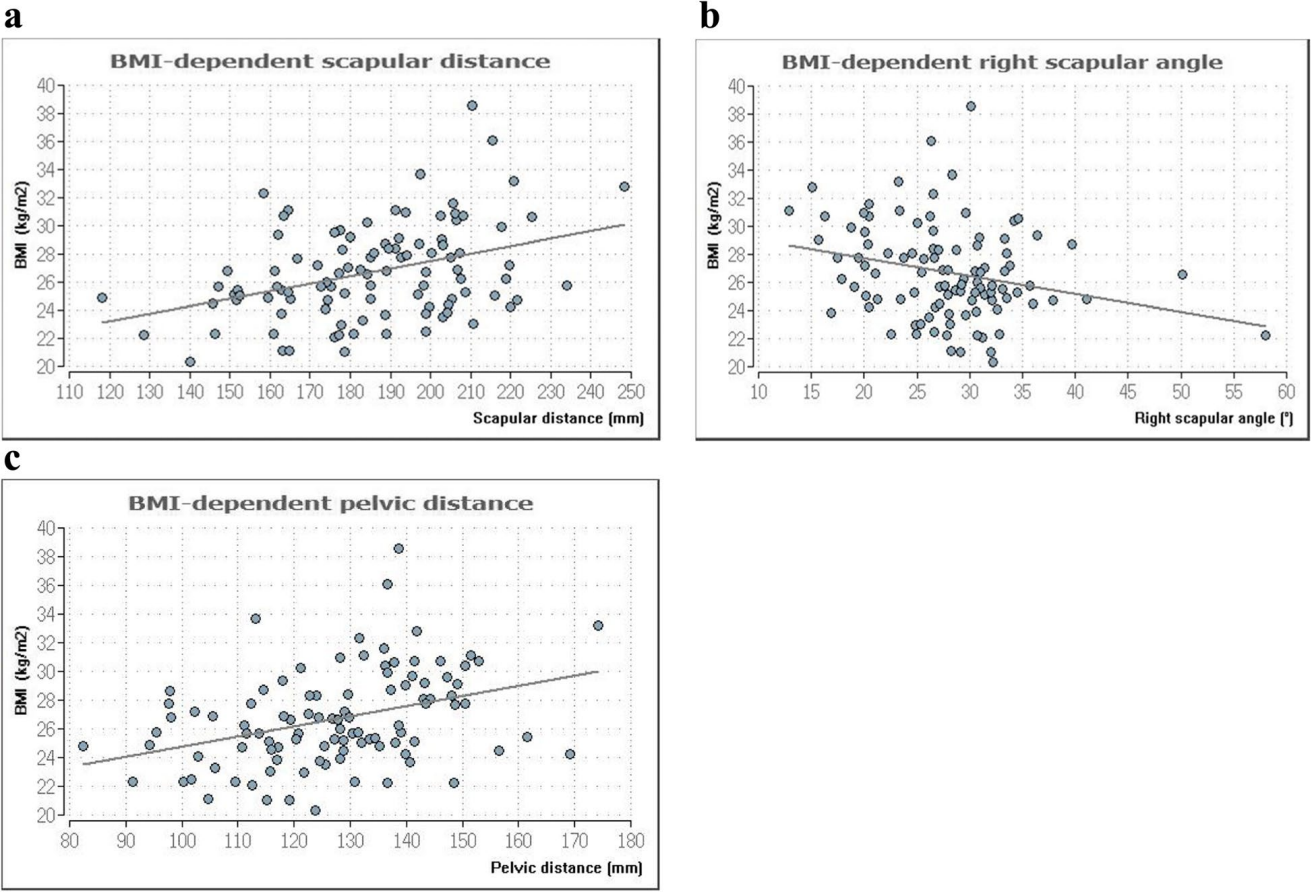
**a**–**c** Significant correlations between the BMI and the scapular distance (**a**), the scapluar angle right (**b**), and the pelvic distance (**c**)

Calculation of the correlations between the upper body static parameters and BMI revealed significant correlations for the pelvic distance DD (*p*≤ 0.001, rho-value = 0.39, effect size weak), scapula distance (*p*≤ 0.001, rho = 0.33, effect size weak), and shoulder stance angle right (*p*≤ 0.01, rho-value = − 0.26, effect size poor) (Fig. 2). All other comparisons were not significant (*p*≥ 0.05).

The division of the BMI into groups (group 1: normal, group 2: pre-adipose, and group 3: obese) according to the WHO definition gave more detailed results. Only significant group differences are listed below (Table 3).

**Table 3:** Comparison of parameters of upper body posture and “BMI,” coding of effect size of eta^2^: 1 = 0.01 small effect, 2 = 0.06 medium effect, 3 = 0.14 large effect. Significant *p* values in bold, shaded gray, and marked with an asterisk

BMI (kg/m^2^)								
Parameter			1. Group normal *n* = 12		2. Group pre-adipose *n* = 72		3. Group obese *n* = 17	
	*p* value	eta^2^	Med.	Min./Max.	Med.	Min./Max.	Med.	Min./Max.
Spine parameter								
Trunk length D (mm)	0.01*	0.10 ^2^	457.85	421.22/510.19	482.71	416.43/555.56	467.66	405.02/539.99
Trunk length S (mm)	0.07	0.05 ^1^	503.63	470.40/554.39	529.55	479.19/597.88	519.96	467.23/606.26
Sagittal trunk decline (°)	0.01*	0.10 ^2^	− 1.29	− 4.96/6.40	− 2.84	− 8.70 /3.75	− 5.55	− 7.67/1.78
Frontal trunk decline (°)	0.26	0.03 ^1^	− 0.81	− 4.22/2.73	− 0.25	− 2.80/2.65	− 0.49	− 3.82/1.22
Axis decline (°)	0.44	0.02 ^1^	− 2.04	− 4.42/2.63	− 0.97	− 5.42/4.10	− 0.84	− 4.07/2.22
Thoracic bending angle (°)	0.56	0.01 ^1^	16.16	6.18/20.22	15.49	5.84/27.92	17.15	9.14/21.32
Lumar bending angle (°)	0.71	0.01 ^1^	11.71	6.31/18.40	11.14	5.48/19.21	10.77	7.86/14.47
Standard deviation Lateral deviation (°)	0.60	0.01 ^1^	4.77	2.12/11.89	4.71	1.21/10.66	4.55	1.44/11.60
Standard deviation Rotation (°)	0.07	0.05 ^1^	3.69	1.09/8.47	3.79	1.40/15.94	2.22	0.73/8.49
*Kyphosis angle* (°)	0.28	0.03 ^1^	52.86	35.97/71.59	51.14	32.30/78.58	55.56	37.52/66.87
*Lordosis angle* (°)	0.31	0.02 ^1^	27.96	18.07/39.52	32.08	19.29/56.34	31.76	19.06/47.54
Pelvis parameter								
Pelvic distance (mm)	0.01*	0.11 ^2^	127.25	102.83/156.56	124.97	82.48/169.19	137.84	113.16/174.12
Pelvic height (°)	0.79	0.01 ^1^	− 0.45	− 3.86/1.63	− 0.65	− 4.90/4.21	− 0.40	− 2.72/2.51
Pelvic height (mm)	0.79	0.01 ^1^	− 0.96	− 9.19/4.44	− 1.55	− 9.46/10.51	− 0.84	− 6.70/5.72
Pelvic torsion (°)	0.99	0.001 ^1^	2.47	− 10.53/5.93	1.69	− 15.99/20.61	1.60	− 15.90/16.70
Pelvic rotation (°)	0.14	0.04 ^1^	− 0.55	− 7.03/6.16	0.91	− 8.52/14.02	− 0.19	− 6.55/2.56
Shoulder parameter								
Scapular distance (mm)	0.01*	0.10 ^2^	174.93	128.67/205.27	184.57	118.17/233.93	205.72	158.31/248.18
Scapular height (°)	0.69	0.01 ^1^	− 6.03	− 15.27/10.36	− 4.05	− 19.79/12.02	− 5.05	− 11.32/14.39
Scapular rotation (°)	0.20	0.03 ^1^	0.40	− 3.37/6.71	1.07	− 5.43/8.16	− 0.13	− 6.41/6.60
Left scapular angle (°)	0.02*	0.08 ^2^	29.94	26.12/35.94	26.31	16.92/51.09	25.83	11.18/71.23
Right scapular angle (°)	0.01*	0.09 ^2^	31.70	26.09/57.99	28.08	15.64/50.10	25.04	12.85/34.33

There was significance (*p*≤ 0.02) in the torso length D (*p*≤ 0.01) between groups 1 and 2. The median of group 1 was 457.85 mm and that of group 2 was 482.71 mm. In addition, there was significance (*p*≤ 0.01) in the sagittal trunk tilt (°) with respect to groups 1 and 3 (*p*≤ 0.01). The median of group 1 was − 1.29° and that of group 3 was − 5.55°. The pelvic distance DD (*p*≤ 0.01) recorded a significant difference (*p*≤ 0.01) between groups 2 and 3, with the median of group 2 being 124.97 mm and that of group 3 being 137.84 mm.

There was also significance (*p*≤ 0.01) in the scapula distance between groups 1 and 3 (*p*≤ 0.01) and groups 2 and 3 (*p*≤ 0.02) (group 1: 174.93 mm, group 2: 184.57 mm, group 3: 205.72 mm). In addition, there was a significant discrepancy (*p*≤ 0.02) in the left shoulder angle. In the group comparison, significances (*p*≤ 0.02) were calculated between groups 1 and 2 and groups 1 and 3 (group 1: 29.94°, group 2: 26.31°, group 3: 25.83°). Furthermore, a significance (*p*≤ 0.01) was calculated for the right shoulder angle between groups 1 and 3 (*p*≤ 0.01; group 1: 31.70°, group 3: 25.04°).

### Physical activity

The comparison of groups with different levels of sporting activity (group 1: rarely/never, group 2: 1x/week, group 3: 2x/week, group 4: > 2x/week) with regard to the upper body posture parameters gave the following significant group differences (*p*≤ 0.01).

The pelvic distance DD was significantly different between groups 1 and 4 (*p*≤ 0.03), with a median of 130.44 mm for group 1 and 120.09 mm for group 4.

**Fig. 3:**
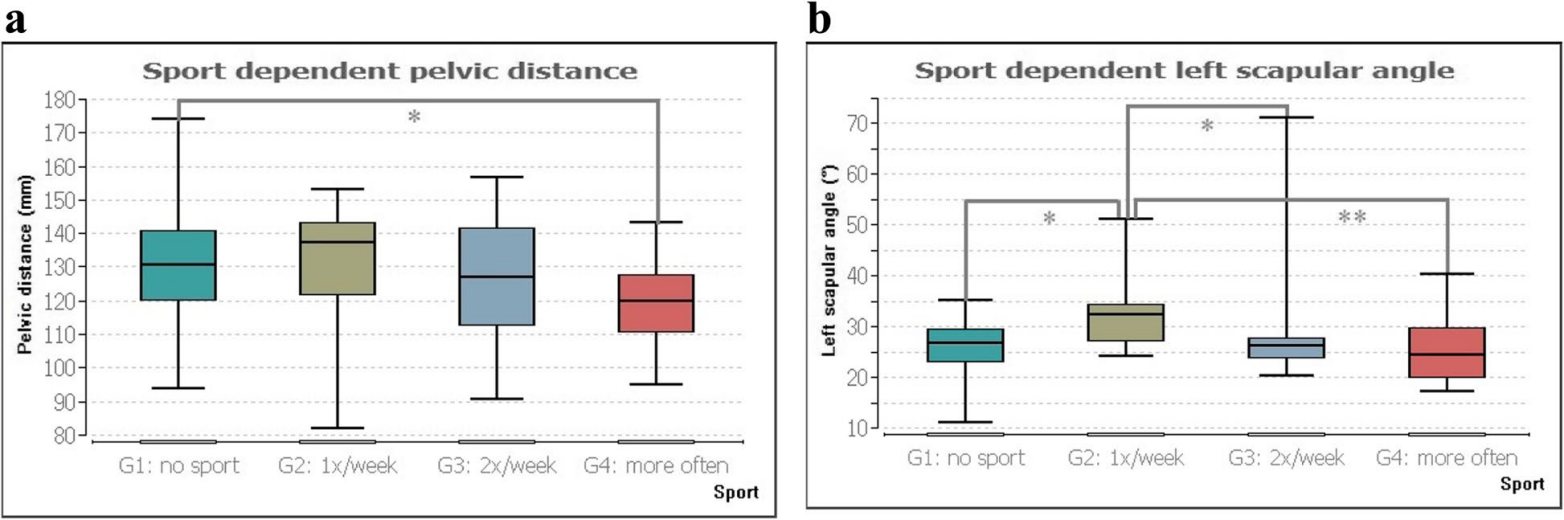
**a** Significant pair comparison of the pelvis distance indicated physical activity. **b** Significant group comparison of the left scapular angle indicated physical activity

There was also significance (*p*≤ 0.01) in the left shoulder angle between groups 1 and 2, 2 and 3, and 2 and 4 (*p*≤ 0.02 and 0.01, respectively). The median of group 1 was 26.60°, for group 2 it was 32.25°, for group 3 26.23° and that of group 4 was 24.50°. Figure 3 illustrates the group differences in the two parameters.

## Discussion

In the present study, an average height of 179.89 ± 7.38 cm with a body weight of 86.36 ± 11.58 kg and a mean BMI of 26.70 ± 3.35 kg/m^2^ were determined for the male subject collective of 101 participants aged 31 to 40 years. Using the WHO classification [8, 16, 17], the BMI results revealed a preadipose majority of 71.29% (72 participants) within the subjects. In contrast, 12 (11.88%) subjects were in the normal range and 17 (16.83%) were obese. Considering the known constitutional data of the German population [21, 22] for men aged 30–40 years, there were no major differences of more than 1 cm, 2 kg, or 1 kg/m^2^. According to Schienkiewitz et al. [23], 61.6% of men in Germany have a BMI of over 25 kg/m^2^ while 43.3% of men have a BMI in the range of 25 to 30 kg/m^2^. Consequently, the constitutional data of the present male subjects can be classified according to these values and can be used as a baseline for the determination of standard values. With regard to the upper body posture, a relatively balanced posture in the spine, shoulder and pelvic area could be determined within the test person collective, whereby the torso was inclined 2.63° ventrally. While the pelvic region was virtually balanced, in the shoulder region, the right shoulder was very slightly more caudal.

The thoracic bending angle was 15.66° and the lumbar bending angle was 10.99°. Equally, the thoracic kyphosis had an average angle of 52.56° and the lumbar lordosis 32.16°. On the basis of the above-mentioned measured values, it can, thus, be concluded that the subjects of the present study have a symmetrical and axis-appropriate posture which can possibly be attributed to the movement system of healthy and symptom-free participants. Therefore, hypothesis 1 can be verified.

Considering the classification of the Scoliosis Research Society [24, 25], normal kyphosis in the thoracic region is between 30 and 50° and a lumbar lordosis between 31 and 79°, whereby it is clear from these degree numbers that the reference range for the lumbar lordosis varies greatly in the literature [24–26]. From this, it can be assumed that the present subject collective generally tended to have a hyperkyphosis with a normally pronounced lordosis. At this point, it must be taken into account that the above-mentioned standard values refer to results taken from radiological measurement procedures. In contrast, both in the present study and in further studies by Ohlendorf et al. [9, 10], the body surface of the participants was recorded via a three-dimensional back scanner (video raster stereography) [11, 19]. Consequently, the smaller the body measurements of the participants, the smaller is the light-irradiated surface. Despite these differences in procedures, the results of both measurement methods differ only slightly and are, thus, comparable with each other under critical evaluation [27, 28].

Compared with the measurement results of the sex-specific studies of other age groups by Ohlendorf et al. [9–12], in which healthy men between 18–35 (*n* = 102) years [9] and 41–50 (*n* = 100) years [10] of age were examined under the same measurement and evaluation conditions, there is, mostly, congruence. The deviations of all parameters from each other are only slight; only the kyphosis angle in the younger men is about 7° lower at 45.85° with an equivalent lordosis angle (30.67°). Accordingly, the kyphosis for the subjects between 18 and 35 years of age is within the normal range [24–26]. As a possible cause for an increased thoracic kyphosis in the present subject collective, Roghani et al. [29] state in their review that age-related factors [30, 31] can not only lead to hyperkyphosis, but also to degenerative diseases, a genetic disposition and a weakly developed deep back musculature. The latter factor, as well as constitutional differences in the sense of a higher BMI and, associated with it, a wider shoulder (+ 6.22 mm) and pelvic distance (+ 33.74 mm) of the markers are most likely the cause. The present participants were, on average, 2.11 cm shorter, 10.36 kg heavier, and had a BMI 3.6 kg/m^2^ higher than the younger age cohort [9]. Similarly, in a study of 41–50-year-old men [10], the distance between the pelvic markers was 34.58 mm less in these participants; this was also due to the constitutional differences of 0.78 cm height, 1.36 kg weight, and 0.70 kg/m^2^ BMI.

BMI, per se, also has an effect on upper body posture. From the significant correlations, it can be concluded that with increasing BMI there is a wider pelvis and also wider scapula distance. In addition, the right shoulder angle increases (meaning more caudally located) with decreasing BMI. As a result, the right shoulder angle becomes more similar to that of the left shoulder angle as the BMI decreases. In the group comparison of the three BMI groups (1) normal, (2) preadipose, and (3) obese, the same findings emerged. The pelvic distance was larger in the obese than in the preadipose, just as the shoulder blade distance was smaller in the normal weight and preadipose than in the obese. Considering the results, it can be speculated that with increasing BMI, the shoulders of the subjects become wider due to the larger body surface area. There are also slight differences between the three BMI groups for the right shoulder position. These differences can also be seen in the group comparison for the right shoulder, but only between normal weight and obese people, with a difference of about 7°. A new finding is that the pre-adipose men were taller than those of normal weight, which is randomly due to the sample size of 101 participants of which 12 men were of normal weight. Furthermore, the obese participants held a stance 4.26° more anteriorly inclined than the normal weight participants. Since males with a higher BMI are inclined further anteriorly (= sagittal trunk inclination; sagittal parameter), hypothesis 2 can also be accepted.

Since the present sample is comparatively heavier, but not necessarily much shorter than other male populations [21–23], weight rather than height is the decisive factor in the observed correlations. Consequently, it can be assumed that as the body weight of the test persons increases, the body surface increases due to the increased fat and muscle content and that this, consequently, also has an effect on the width of the shoulder blades and the pelvis. In this context, it must be taken into account that the present measurement procedure is a representation of the dorsal surface, i.e. the dorsal surface structure. By means of previously glued landmarks, triangulatory calculations are then performed. For example, the “pelvis distance” and “scapula distance” are based on distance measurements between the left and right marker. A stronger stature will result in an increase of these distances in view of the regular geometric relations between the dorsal surface and the body shape.

In men, increased body weight often results in an associated forward curvature of the abdomen, an anteriorly shifted center of gravity, an increasing thoracic kyphosis and also an increasing lumbar lordosis.^32^ Since the effect sizes of the correlations with regard to the anamnestic parameters for body weight and BMI are in the weak range, this result should be regarded rather as a tendency. However, why the increased weight in the present subjects is only visible in the thoracic kyphosis area and not additionally in the lumbar area of the lordosis must be investigated in future studies and cannot be explained on the basis of the available data.

Considering the pair comparisons with regard to the physical activity of the test persons (group 1: rarely/never, group 2: 1x/week, group 3: 2x/week, group 4: > 2x/week), it was discovered that subjects without physical activity had a pelvis that was approximately 10 mm wider than those participants who undertook physical activity more than twice a week. Furthermore, the sportier the group, the more caudally the left shoulder was positioned. A more detailed examination revealed that the subjects who do not exercise were statistically thicker in relation to their weight (median: 89 kg) than those who exercise more than twice a week (median: 84 kg). Similarly, the median BMI was found to decrease with increasing physical activity. These data confirm the assumption made above that the distances between the pelvis and shoulder blades decrease with less body mass. This clearly shows that there is a correlation between body weight and BMI in relation to upper body posture, based on the available results of the study. Consequently, hypothesis 3 has to be falsified.

When carrying out the three-dimensional measurements of the upper body posture, however, it should be borne in mind that limitations should be taken into account. For example, highlighted spots due to singular light rays or reflective hair accessories (which should be removed before the scan) can falsify the measurement. In addition, extensive dark areas, such as large tattoos or shadows caused by excessive skin folds, can also affect the measurement process. Apart from that, the measurement of upper body posture was performed in habitual standing position. It would also be interesting to conduct an analysis during movement, as incorrect postures could possibly come to light here. Handedness has also been left out of this analysis. Since approx. 95% of the participants were right-handed, handedness was not considered as an analysis factor. Furthermore, external influences (occupational environment) were not assessed which might influence the body posture. Besides, the placing of the reflective markers must be taken into consideration, as these can also affect the outcome. However, in this study, the placing was performed according to a standardized procedure [13] by trained examiners. A comparison of the available data with X-ray images with regard to possible agreement of selected parameters, such as the lordosis or kyphosis angle, would allow an intermediate therapeutic diagnosis without radiological radiation. This investigation would be desirable, among other things, with regard to a comparison of measurement systems, since videorasterstereography only records the superficial structures of the back via light projection [14] and radiological measurements, as an imaging method, can depict structures internal to the body. Thus, two completely divergent methods would have to be compared. Additionally, the deviation in height and body weight in our group from other reference values warrants a confirmation of our data in further studies.

In summary, we succeeded in generating quantitative standard reference values of upper body dorsal posture of a large number of subjectively declared healthy males of the age group 30-41 years (*n*= 101) using a non-invasive measurement method (videorasterstereography) with a high (intra- and inter-day) reliability and reproducibility [14]. Thus, the present results complete the previously existing data gap in reference values for this age. The incorporation of the present data into already published standard reference values of other male age groups [9, 10] shows congruence. This topic is in line with an important public health issue of increasing problems in musculoskeletal systems due to modern life style.

## Conclusion

Within the scope of this observational study on the upper body posture of men between 31 and 40 years of age in Germany, it can be concluded that they have a symmetrical body posture which is in line with the axis. Only the thoracic kyphosis is more pronounced than in younger or older men, which, in comparison, can be attributed to a larger BMI or more body weight and, thus, more upper body fat. This can be confirmed by the fact that the shoulder and pelvic distance correlates with athleticism, according to which these distances are smaller in more athletically active men of this age group.

## Data Availability

The datasets supporting the conclusions of this article are included within the article.

## Abbreviations

BMI: Body mass index
ICD: International Statistical Classification of Diseases and Related Health Problems
TR: Tolerance range
CI: Confidence interval
